# Platelet count/bipolar spleen diameter ratio for the prediction of esophageal varices: The special Egyptian situation: Noninvasive prediction of esophageal varices

**Published:** 2011-04-01

**Authors:** Mona A. Abu El Makarem, Mohamed E. Shatat, Yehya Shaker, Ahmad A. Abdel Aleem, Ali M El Sherif, Maysa Abdel Moaty, Hosny S. Abdel Ghany, Atef Elakad, Amal M. Kamal Eldeen

**Affiliations:** 1 Department of Internal Medicine, Minia University, Minya, Egypt; 2 Department of Radiology, Minia University, Menia, Egypt; 3 Department of Clinical pathology, Minia University, Minya, Egypt

**Keywords:** Cirrhosis, Platelet count, Bipolar disorders, Spleen

## Abstract

**Background:**

Esophageal variceal hemorrhage is a devastating complication of portal hypertension that occurs in approximately one-third of cirrhotic patients.

**Objectives:**

We assessed the value of the platelet count/ bipolar spleen diameter ratio as a noninvasive parameter for the prediction of esophageal varices (EVs) in Egyptian cirrhotic patients.

**Patients and Methods:**

Laboratory and ultrasonographic and imaging variables were prospectively evaluated in 175 patients with liver cirrhosis. All patients underwent upper gastrointestinal endoscopy. Patients with active gastrointestinal bleeding at the time of admission were excluded.

**Results:**

The platelet count/ bipolar spleen diameter ratio in patients with EVs was significantly lower than in patients without EVs. In an analysis of the receiver operating characteristic curves (ROCs), we calculated an optimal cutoff value of 939.7 for this ratio, which gave 100% sensitivity and negative predictive values, 86.3% specificity, a 95.6% positive predictive value, and an area under the ROC curve of 0.94 ± 0.02, reflecting its overall diagnostic accuracy. These findings were extended to a subset analysis of compensated cirrhotic patients.

**Conclusions:**

The platelet count/ bipolar spleen diameter ratio has excellent accuracy in the noninvasive assessment of EVs in patients with compensated or decompensated liver cirrhosis. It is easy to calculate and can lower the financial and sanitary burdens of endoscopy units, especially in developing countries.

## Background

Portal hypertension and esophageal varices (EVs) are common major complications of liver cirrhosis, occurring in approximately 24% to 80% of cases, with an extremely high mortality rate [1][2][3]. Therefore, the prevention of variceal bleeding is an important goal in management patients with liver cirrhosis. Universal endoscopic screening of EVs is recommended in conjunction with primary prophylaxis in patients who are at high risk of variceal bleeding [4][5]. But, this screen is invasive, and many patients will not have varices, rendering this method cost-ineffective. Thus, noninvasive diagnosis of portal hypertension may be useful [2]. Recently, several studies have attempted to identify the variables that can predict the presence of EVs-even large EVs-noninvasively, examining various biochemical, clinical, and ultrasonographic parameters alone or in combination, with promising results overall [6][7][8][9].

Most such variables, however, have several limitations, which has hindered the wide application of these results. Early studies were retrospective and were performed in a specific subgroup of patients-those who were going to be placed on a wait list for liver transplantation [8][10][11][12][13][14]; thus, the study groups lacked homogeneity and the wide representation of the cirrhotic population that is seen in clinical practice. Further, in patients with chronic liver disease, although the presence of thrombocytopenia is due primarily to portal hypertension [15], thrombocytopenia can depend on other factors, such as shortened mean platelet lifetime, decreased thrombopoetin production, and the myelotoxic effects of alcohol or hepatitis viruses [16]; thus, the autoimmune profile and bone marrow aspirate should be assessed for greater accuracy before conclusions can be made on the final data that thrombocytopenia is owing to liver cirrhosis.

Finally, there has been a lack in uniformity in the classification and diagnosis of EVs in previous studies [8][10][11][12][13][14], in which EVs were not categorized by a single endoscopist or in the same endoscopy unit. Moreover, their focus on patients with large EVs might have led to the omission of an important subset of patients who required medical counseling; thus, the analysis of the presence or absence of EVs might prevent data from being misinterpreted and allow results to be generalized [15]. The platelet count:spleen diameter ratio, proposed by Giannini et al. [15], appears to be one of the best noninvasive predictors of EVs that have emerged [17].

## Objectives

In this prospective study, we evaluated the platelet count:spleen diameter ratio in 175 consecutive unselected Egyptian cirrhotic patients-with varying ethnicities and clinical presentations and poor nutritional status, many of whom had a viral etiology-in predicting EVs.

## Patients and Methods

### Eligible patients

This study included 175 consecutive patients with liver cirrhosis due to hepatitis C virus. Liver cirrhosis was diagnosed by physical, laboratory, and radiological evaluations. Diagnosis was confirmed by histological examination of Tru-cut needle-isolated liver biopsy for patients with Child-Pugh class A. After written consent was obtained, the participants underwent upper GIT endoscopy in our endoscopy unit, Minia University Hospital, between April 2008 and March 2010. The exclusion criteria were: active variceal bleeding at admission, a history of endoscopic variceal sclerotherapy or band ligation, transjugular intrahepatic portosystemic stent shunt placement, a history of surgery for portal hypertension, medication use for primary prophylaxis of variceal bleeding, alcohol abuse, and thrombocytopenia due to causes other than hypersplenism. Other complications of liver cirrhosis were not exclusion criteria, although they were recorded.

### Informed consent

The study protocol was approved by the Institutional Ethics Committee of the School of Medicine, Minia University, Egypt, and all patients gave their informed consent to participate. The study was conducted in accordance with the ethical guidelines of the 1975 Declaration of Helsinki and the International Conference on Harmonization Guidelines for Good Clinical Practice.

### Clinical and Laboratory Assessment

At the time of the upper endoscopy, a history was taken and a clinical examination was performed, with special emphasis on the stigmata of chronic liver disease and a careful abdominal examination. After hospital admission, venous blood was drawn to determine the complete blood count, prothrombin time and concentration, liver function, and renal function. In thrombocytopenic patients (platelets < 140,000/cmm), anti-nuclear antibody (ANA), anti-smooth muscle antibody (ASMA), and gamma-globulin levels were measured and bone marrow aspiration was performed to exclude causes of thrombocytopenia other than portal hypertension.

### Imaging Study

Abdominal ultrasonography: Cirrhosis was diagnosed per the criteria of Tchelepi et al. [18]. Maximum bipolar spleen diameter was measured by ultrasonography and expressed in millimeters per Lamb et al. [19]. The platelet count/ bipolar splenic diameter ratio was calculated for all patients. Duplex study of the portal vein: Mean portal blood flow velocity (MPBV) was measured in the portal vein trunk per Mori Yasu et al. [20] and expressed in the Duplex Doppler system as V mean. After these evaluations were completed, all cirrhotic patients were classified per Child-Pugh&apos;s criteria [21], and their scores were calculated.

### Upper Endoscopy

Patients were evaluated for the presence of EVs, gastropathy, and other findings. All endoscopies were performed by a single expert endoscopist who was blinded to the patient's data.

### Statistical analysis

All data were tabulated. SPSS® (USA) version 11 was used for the statistical analysis. A descriptive analysis was performed for all data. Numerical data were expressed as mean ±SD and range, and categorical data were expressed as number and percentage. T-test was used to compare 2 independent groups of data. Chi-square test was used to compare categorical groups of data. Multivariate logistic regression analysis was performed on parameters that differed significantly in the univariate analysis between patients with no EVs and those with EVs to determine the variables that were independently associated with the presence of EVs. Receiver operating characteristic (ROC) curves were generated to determine the cutoff values for the best sensitivity and specificity of the variables with regard to the presence of EVs. Also, the ROC curves were used to identify the cutoff prevalence-adjusted negative and positive predictive values for the presence of EVs. The validity of the model was determined using the concordance (c) statistic (equivalent to the area under the ROC curve). A model with a c-value above 0.7 is considered to be useful, and a c-value between 0.8 and 0.9 indicates excellent diagnostic accuracy. A P-value was considered to be nonsignificant if > 0.05 and significant if ≤ 0.05.

## Results

### Frequency of EVs and Correlation with Clinical, Laboratory, and Radiology Findings

One hundred fifteen men and 60 women were included in the study. The mean age was 48 years (range 36-60 years). Forty-six patients were Child-Pugh class A (26.3%), 59 were class B (33.7%), and 70 were class C (40%). EVs were detected in 131 patients (74.9%). Further, EVs were observed in 14 (30.4%) in the 46 patients with compensated cirrhosis. The presence of EVs correlated significantly with the severity of liver cirrhosis (p = 0.001), as measured by Child-Pugh score (Table 1). Patients with EVs were older; had lower platelet counts; higher Child-Pugh scores; developed hepatic encephalopathy, ascites, and jaundice more frequently; and had lower prothrombin concentrations, lower platelet count/bipolar spleen diameter ratios, and higher bipolar spleen diameters (Tables 1, Tables 2, Tables3). Although patients with EVs had higher portal vein diameter PVDs and mean portal blood velocity, these findings were not significant (p = 0.07, p = 0.9; respectively) (Table 3).

**Table 1:** Distribution of patients with and with esophageal varices by Child-Pugh score

**Variables**	**Patients with EVs a** (No. = 131) (74.9%)	**Patients without EVs a**(No. = 44) (25.1%)	**p-value**
**Child A**	14 (10.7%)	32 (72.7%)	0.001
**Child B**	52 (39.7%)	7 (15.9%)	
**Child C**	65 (49.6%)	5 (11.4 %)	

^a^ EVs = esophageal varices. Data are expressed as numbers and percentages and compared by chi-square test.

**Table 2:** Demographic, clinical, and laboratory characteristics of cirrhotics with and without esophageal varices

**Variables**	**Cirrhotics with EVs a**(No. = 131)	**Cirrhotics without EVs a**(No. = 44)	**p-value**
**Age** (years)	51.09 ± 5.1	46.8 ± 7.9	0.001
**Sex**			
**Male** (%)	83 (63.4 %)	32 (72.7 %)	0.2
**Female** (%)	48 (36.6 %)	12 (27.3%)	
**Splenomegaly** (%)	131 (100%)	44 (100 %)	-
**Child-Pugh score**	9.8 ± 2.8	6.8 ± 2.3	0.001
**Platelet count **(No/cmm)	119480.9 ± 38725.7	213000 ± 69232.6	0.001

^a^ EVs = esophageal varices

**Table 3:** Characteristics of imaging study in cirrhotics with and without esophageal varices

**Variables**	**Cirrhotics with EVs^c^**(No. =131)	**Cirrhotics without EVs c**(No. = 44)	**p-value**
**PVD a** (mm)	13.04 ± 1.9	12.4 ± 2.0	0.07
**Bipolar spleen diameter **(mm)	159.4 ± 24.2	140.5 ± 20.7	0.001
**Platelet count/bipolar spleen diameter ratio**	747.6 ± 197.6	1588.8 ± 744.9	0.001
**MPBV b**	12.8 ± 2.2	12.8 ± 2.8	0.9

^a^ PVD = portal vein diameter

^b^ MPBV = mean portal blood velocity

^c^ EVs = esophageal varices

### Factors Associated with EVS by Multivariable Analysis

**Figure 1:**
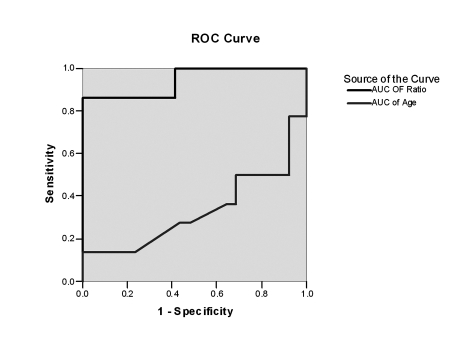
Receiver operating characteristic curve (roc) of platelet count/ bipolar spleen diameter ratio and age, AUC = area under the curve, AUC for ratio = 0.94 ± 0.02, AUC for age = 0.33 ± 0.05

In the multivariate ordinal regression analysis, the presence of EVs was associated significantly with platelet count: spleen diameter ratio (odds ratio; 1.028, 95% CI: 1.016-1.04, P = 0.001) and age (odds ratio; 1.205, 95% CI: 1.043-1.392, P = 0.01) (Table 4). The area under the ROC curve for the platelet count/ bipolar spleen diameter ratio was 0.94 ± 0.02 (Figure 1), which represents the overall diagnostic accuracy of the ratio in predicting EVs. Thus, on random selection of an individual with EVs versus no EVs, the score of the former will be lower 94% of the time. Table 5 shows the sensitivity, specificity, PPV, and NPV of the ratio at various cutoff levels. At a score of 939.7, the ratio has high sensitivity (100%), robust negative predictive value NPV (100%), and high specificity (86.3%) and positive predictive value PPV (95.6%).

In a separate analysis, the platelet count/bipolar spleen diameter ratio was significantly higher in compensated cirrhotics (Child-Pugh class A) without EVs compared with those with EVs (1802 ± 987 and 770 ± 146.6, respectively; p = 0.0001). In this analysis, the ratio maintained high sensitivity (100 %), robust NPV (100%), and high specificity (81.8%) and PPV (97%), with an overall diagnostic accuracy of 87.2% (Table 6). Notably, none of the thrombocytopenic patients showed any evidence of thrombocytopenia for etiologies other than hypersplenism, as evidenced by the normal results for ANA, ASMA, gamma-globulin levels and bone marrow aspiration (data not shown).

**Table 4:** Best-fitting multiple logistic regression predictors of esophageal varices

** Predictor **	** Regression coefficient **	** Odds ratio **	** 95% CI **	** p-value **
** Platelet count/bipolar spleen diameter ratio **	0.633	1.028	1.016-1.041	0.001
** Age **	0.150	1.205	1.043-1.392	0.01

**Table 5:** Predictive accuracy of the best cutoff value of platelet count/ bipolar spleen diameter ratio in the diagnosis of esophageal varices

**Cutoff value**	**Sensitivity**	**Specificity**	**NPV a**	**PPV b**	**Accuracy**
939.7	100%	86.3%	100%	95.6%	96.5%

^a^ NPV = negative predictive value

^b^ PPV = positive predictive value

**Table 6:** Validation of cutoff value of platelet count/ bipolar spleen diameter ratio in the diagnosis of esophageal varices in patients with compensated liver cirrhosis

**Sensitivity**	**Specificity**	**NPV a**	**PPV b**	**Accuracy**
100%	81.8 %	100 %	70 %	87.2 %

^a^ NPV = negative predictive value

^b^ PPV = positive predictive value

## Discussion

Management of EVs is an everyday challenge. Current guidelines recommend that all patients should undergo endoscopic screening for varices when cirrhosis is diagnosed, after which patients with medium and large varices should be treated to prevent bleeding. For all other patients, regular periodic evaluation is required [22]. In Egypt, however, the management of patients with liver cirrhosis complicated by the interplay between clinical, economic, social, and cultural factors and the generally poor compliance to both follow-up and treatment strategies. Endoscopic follow-up, which is recommended by international guidelines, is not feasible in most patients for many reasons. Liver diseases are common in Egypt due to the higher prevalence of viral hepatitis [23][24] and increased incidence of schistosomiasis [25]; moreover, most patients present in the late phase of liver disease. Further, the majority of patients is uninsured and must pay for expenses out of pocket, unaware of the risk of the variceal bleeding, and they are apparently healthy asymptomatic compensated patients. Compliance can also be limited, because it requires that patients who are asymptomatic undergo a procedure repeatedly that is perceived to be unpleasant.

Endoscopic follow-up is also impractical due to the fear of infection; despite efforts to ensure sterilization, there remains a risk of re-infection by another subtype of the same virus infect the patient or another viral hepatitis with increased risk of decompensation in compensated patients [26]. Finally, endoscopy units are not available in all hospitals, particularly in rural areas, necessitating other easier modalities for the diagnosis and monitoring of portal hypertension. Thus, a method of predicting the presence of EVs noninvasively is in great demand to avoid unnecessary endoscopy and improve the cost-effectiveness of management; the latter is a particularly important consideration in many African and Middle Eastern countries, including Egypt, where liver cirrhosis is highly prevalent. Ideally, a method for identifying patients with varices should be simple, noninvasive, inexpensive, reproducible, accurate, and readily available; have high sensitivity and specificity; follow the natural history; reflect the effect of the treatment accurately; and indicate the prognosis and possibility of the success of a treatment. Several noninvasive and minimally invasive methods have emerged in recent years, assessing the potential of various laboratory, clinical, and ultrasonographic parameters, linked directly or indirectly to portal hypertension, such as splenomegaly, decreased platelet count, and portal vein diameter [8][9][10][22][27][28][29][30][31]. Three such methods have been examined extensively: platelet count:spleen diameter ratio [15], Fibrotest [32], and Fibroscan [33]. Fibrotest appears to be insufficiently precise, and Fibroscan requires further evaluation; neither test is widely available in Egypt due to financial and technical considerations. In a seminal trial, Giannini and colleagues used the platelet count:spleen diameter ratio as a parameter, linking thrombocytopenia to spleen size to predict portal hypertension [15]. Although these international trials; the differences in the populations characters and the need to a well-designed repeated multiple large multi-central trials, before any final recommendation can be concluded, lead to that the conclusions of the Baveno IV consensus workshop on portal hypertension were that, endoscopic screening has been the optimal method of detecting varices [4][5]. For these reasons, we planned the current study to evaluate the use of a similar ratio in Egyptian cirrhotic patients who typically present late and have varying ethnicities, poorer nutritional status, and significant viral etiology to overcome the drawbacks of previous studies. (We think that this paragraph doesn't need modification).

In our study, the prevalence of EVs was 74.9%, which might be attributed to the late presentation of our patients and the increased incidence of schistosomiasis in our region [25]. In addition to age, serum bilirubin, prothrombin activity, platelet count, spleen diameter, and Child-Pugh scores, the platelet count:spleen diameter ratio was associated with portal hypertension. These results were confirmed by multivariate logistic regression, which demonstrated that the platelet count: spleen diameter ratio (odds ratio: 1.028, 95% CI: 1.016-1.04, P = 0.001) and age were independent predictive factors for EVs (odds ratio: 1.205, 95% CI: 1.043-1.392, P = 0.01). Although portal vein diameter, as assessed by ultrasonography, was higher in cirrhotic patients with EVs, this correlation failed to reach statistical significance (P = 0.07), as did mean portal blood flow velocity; these data are consistent with results of other large trials [8][10][11][12][13][14]. Moreover, performing Doppler sonographic examination requires sophisticated skills and equipment, limiting its value in the identification of patients with cirrhosis who are at risk of variceal bleeding [34]. Our analysis of the area under the ROC curve (AUROC) revealed that the cutoff of the platelet count:spleen diameter ratio (939.7) was the optimal value for accurate prediction of EVs with an AUC of 0.95; this value corresponded to positive and negative predictive values of 96% and 100%, respectively. It has been reported that a negative predictive value (NPV) of 100% is desirable for screens to minimize the oversight of individuals who are at risk [13][17]. This finding is consistent with the initial study by Giannini et al. [15], who used a cutoff of 909 with an AUROC curve of 0.981, corresponding to positive and negative predictive values of 95.6% and 100%, respectively, for the presence of varices. The same ratio has also been examined by many groups in many countries in patient populations that differed from the group in which it was developed, generating consistent results-suggesting that the ratio is generalizable [35][36][37][38]. We believe that this ratio is valuable and unique-a hypothesis that is supported by many clinical, financial, and statistical findings. Clinically, the increase in spleen size in patients with chronic liver disease is nearly always a manifestation of portal hypertension [39][40]; conversely, although thrombocytopenia can result from immune-mediated mechanisms or lower thrombopoietin synthesis [16][41], in most cases it is usually caused by splenic pooling of platelets due to portal hypertension [15][42]. This model is supported by our results, in which thrombocytopenia was attributed to hypersplensim in all patients. Integrating platelet count and spleen size in a ratio allowed us to determine the extent of thrombocytopenia that most likely resulted from hypersplenism. Financially, the ratio is easy to calculate and can be used at the bedside, Biannual calculation of the ratio will not generate additional costs in the management of cirrhotic patients, because platelet count is assessed routinely and abdominal ultrasonography is usually performed at least semiannually to monitor hepatocellular carcinoma [43]. In fact, spleen bipolar measurements consistently show high reproducibility and low intra- and interobserver variability [44][45].

Statistically, the platelet count:spleen diameter ratio and age were the only parameters that were independently associated with the presence of EVs in the multivariate analysis. The AUROC curves for age and platelet count:spleen diameter ratio were 0.31 and 0.95, respectively, indicating that the ratio can be used as the sole predictive factor for EVs. Notably, in a subset analysis, this ratio remained valuable in the prediction of the presence of EVs in patients with no signs of decompensation, with 100% sensitivity and NPV. This property might be particularly useful; its clinical importance has recently been emphasized [3][10][46][47]. Although our results were based on a subset analysis and in smaller sample sizes, routine periodic endoscopy, as recommended by the Ministry of National Health in Egypt, should be considered in the follow-up of cirrhotic patients; based on limitations in financial resources, this sample ratio; platelet count:spleen diameter ratio should be applied.

In conclusion, the platelet count:spleen diameter ratio is an accurate noninvasive method of assessing EVs in Egyptian patients with compensated or decompensated liver cirrhosis. It is easy to calculate and can reduce the financial and sanitary burdens of endoscopy units, particularly in developing countries. Additional large multicenter studies on this ratio should be performed.
